# Novel Phosphorylation-State Specific Antibodies Reveal Differential Deposition of Ser26 Phosphorylated Aβ Species in a Mouse Model of Alzheimer’s Disease

**DOI:** 10.3389/fnmol.2020.619639

**Published:** 2021-01-15

**Authors:** Sathish Kumar, Akshay Kapadia, Sandra Theil, Pranav Joshi, Florian Riffel, Michael T. Heneka, Jochen Walter

**Affiliations:** ^1^Department of Neurology, University of Bonn Medical Center, Bonn, Germany; ^2^Department of Neurodegenerative Diseases and Geropsychiatry, Neurology, University of Bonn Medical Center, Bonn, Germany; ^3^German Center for Neurodegenerative Diseases (DZNE), Bonn, Germany

**Keywords:** Alzheimer’s disease, amyloid-β peptide, cerebral amyloid angiopathy, post-translational modification, modified amyloid-β, phosphorylation, monoclonal antibody, mouse models

## Abstract

Aggregation and deposition of amyloid-β (Aβ) peptides in extracellular plaques and in the cerebral vasculature are prominent neuropathological features of Alzheimer’s disease (AD) and closely associated with the pathogenesis of AD. Amyloid plaques in the brains of most AD patients and transgenic mouse models exhibit heterogeneity in the composition of Aβ deposits, due to the occurrence of elongated, truncated, and post-translationally modified Aβ peptides. Importantly, changes in the deposition of these different Aβ variants are associated with the clinical disease progression and considered to mark sequential phases of plaque and cerebral amyloid angiopathy (CAA) maturation at distinct stages of AD. We recently showed that Aβ phosphorylated at serine residue 26 (pSer26Aβ) has peculiar characteristics in aggregation, deposition, and neurotoxicity. In the current study, we developed and thoroughly validated novel monoclonal and polyclonal antibodies that recognize Aβ depending on the phosphorylation-state of Ser26. Our results demonstrate that selected phosphorylation state-specific antibodies were able to recognize Ser26 phosphorylated and non-phosphorylated Aβ with high specificity in enzyme-linked immunosorbent assay (ELISA) and Western Blotting (WB) assays. Furthermore, immunofluorescence analyses with these antibodies demonstrated the occurrence of pSer26Aβ in transgenic mouse brains that show differential deposition as compared to non-phosphorylated Aβ (npAβ) or other modified Aβ species. Notably, pSer26Aβ species were faintly detected in extracellular Aβ plaques but most prominently found intraneuronally and in cerebral blood vessels. In conclusion, we developed new antibodies to specifically differentiate Aβ peptides depending on the phosphorylation state of Ser26, which are applicable in ELISA, WB, and immunofluorescence staining of mouse brain tissues. These site- and phosphorylation state-specific Aβ antibodies represent novel tools to examine phosphorylated Aβ species to further understand and dissect the complexity in the age-related and spatio-temporal deposition of different Aβ variants in transgenic mouse models and human AD brains.

## Introduction

Alzheimer’s disease (AD) is the most common form of dementia worldwide (Alzheimer’s Association). The two primary pathological hallmarks of the AD brain are abnormal extracellular deposits of amyloid-β (Aβ) peptide and intracellular neurofibrillary tangles (NFTs) of tau protein (Selkoe and Hardy, 2016; Goedert, 2018; DeTure and Dickson, 2019; Jellinger, 2020). The aggregation and deposition of Aβ peptides in the form of amyloid plaques is a critical early step in the disease process that is hypothesized to trigger a complex pathological cascade that ultimately leads to the development of clinical dementia (Duyckaerts et al., 2009; Braak et al., 2011; Calderon-Garcidueñas and Duyckaerts, 2017; Davidson et al., 2018). The critical role of Aβ in the pathogenesis of AD is strongly supported by the identification of early-onset familial AD (FAD)-causing mutations within the genes encoding either the amyloid precursor protein (APP) itself or presenilin 1 and 2 (PS1 and PS2) that commonly alter the production of Aβ peptides in quantitative and qualitative ways (Bateman et al., 2011; Benilova et al., 2012; Katsnelson et al., 2016; De Strooper and Karran, 2016). Strikingly, mutations identified in the APP either within or close to the Aβ region affect Aβ production or alter Aβ aggregation properties, and thereby promote the formation of toxic Aβ aggregates (Grant et al., 2007; Hunter and Brayne, 2018). Further, there is strong evidence that the genetic risk for AD that has been associated with polymorphisms in both Apoliprotein E (ApoE) and Clusterin (CLU) is at least partly attributable to effects of these proteins on Aβ deposition (Ray et al., 1998; Tanzi, 2012; Bettens et al., 2013; Karch et al., 2014; Tcw and Goate, 2017; Belloy et al., 2019). Collectively, these genetic studies indicate that the accumulation and aggregation of Aβ can be a trigger in the pathogenesis of AD-related dementia.

Amyloid deposits in the parenchyma and vasculature consist mainly of Aβ peptides with 38–43 amino acids (Aβ38, Aβ40, Aβ42, and Aβ43; Masters et al., 1985; Glenner and Wong, 2012; Moro et al., 2012). Consistent with an increased propensity to form aggregates (Harper and Lansbury, 1997; Rochet and Lansbury, 2000; Lansbury and Lashuel, 2006; Grant et al., 2007; Teplow, 2012), Aβ42 is the predominant species found initially in amyloid plaques in the parenchyma (Iwatsubo et al., 1996; Mann and Iwatsubo, 1996). In addition to these well-known amino acid length variants, several additional N- and C-terminally truncated or elongated Aβ variants have been described (Saido et al., 1996; Russo et al., 1997; Tekirian et al., 1998; Geddes et al., 1999; Saito et al., 2011; Schönherr et al., 2016; Becker-Pauly and Pietrzik, 2017; Dunys et al., 2018; Walter et al., 2019). Further heterogeneity in Aβ species comes from several post-translational modifications that are also found in characteristic Aβ deposits in parenchymal extracellular plaques and cerebral amyloid angiopathy (CAA; Saido et al., 1995; Shimizu et al., 2000; Milton, 2001, 2005; Miravalle et al., 2005; Schilling et al., 2008; Wirths et al., 2010; Kumar et al., 2011, 2016; Kummer et al., 2011; Frost et al., 2013). These truncated, elongated, and post-translationally modified Aβ peptides have peculiar characteristics in aggregation behavior, deposition, and biostability (Kumar and Walter, 2011; Bayer and Wirths, 2014; Thal et al., 2015; Barykin et al., 2017; Roher et al., 2017; Wirths and Zampar, 2019).

The phosphorylation at Serine residue 8 promotes aggregation, increases neurotoxicity and affects stability and proteolytic degradation of Aβ (Kumar et al., 2011, 2012; Rezaei-Ghaleh et al., 2016a, b). By using phosphorylation-state specific monoclonal antibodies (mAbs), we showed that phosphorylated Ser8-Aβ (pSer8Aβ) accumulates early inside of brain neurons of transgenic APP/PS1 knock-in mice, and is also a prominent component of extracellular plaques during aging (Kumar et al., 2013). The presence of pSer8Aβ was also demonstrated in the brains of human sporadic AD, FAD, CAA, Down syndrome (DS) cases, non-human primates, canines, and a variety of transgenic mouse models (Kumar et al., 2011, 2018, 2020; Rijal Upadhaya et al., 2014; Ashby et al., 2015; Gerth et al., 2018). Aβ can also undergo phosphorylation at serine residue 26, which strongly affects its conformation, aggregation, neurotoxicity, and deposition (Milton, 2001; Kumar et al., 2016). Importantly, Ser26-phosphorylated Aβ (pSer26Aβ) species assemble into specific oligomeric forms that do not proceed further into larger fibrillar aggregates (Rezaei-Ghaleh et al., 2014; Rezaei-Ghaleh et al., 2016b). pSer26Aβ occurs *in vivo* in transgenic mouse models of AD and in human AD brains, showing contrasting distribution as compared to non-phosphorylated Aβ (npAβ) peptides. Furthermore, phosphorylation of Aβ at Ser26 strongly promotes the formation and stabilization of low molecular weight soluble Aβ oligomers with increased toxicity on human neurons (Kumar et al., 2016).

In the current study, we developed and thoroughly validated novel site- and phosphorylation-state Ser26-Aβ specific antibodies. Our results show that selected phosphorylation state-specific antibodies recognize Ser26-phosphorylated and non-phosphorylated Aβ with high specificity in enzyme-linked immunosorbent assay (ELISA), Western Blotting (WB), and immunofluorescence staining. Our study reveals the selective accumulation of pSer26Aβ species in cerebral blood vessels and intraneuronal deposits in transgenic mouse brains.

## Materials and Methods

### Reagents and Antibodies

Synthetic non-modified and post-translationally modified Aβ1–40 and Aβ1–42 peptides were purchased from Peptide Specialty Laboratory (Germany). Methanol was from Sigma. Precast 4–12% NuPAGE Bis-Tris mini gels and prestained protein molecular weight markers were from Life technologies. Nitrocellulose membranes were from Schleicher and Schuell (Germany). WB detection reagents were from GE Healthcare (UK) or LiCOR Biosciences. Protease and phosphatase inhibitors were from Roche Laboratories (Germany). BCA^TM^ protein assay kit was from Thermo Fisher Scientific (USA). Mouse monoclonal Aβ antibodies 6E10 and 4G8 were purchased from Covance Laboratories (USA), and 82E1 antibody was from IBL Corporation (Japan). Development and application of rat monoclonal 7H3D6 antibody were described previously (Kumar et al., 2013). The anti-mouse, anti-rabbit secondary antibodies conjugated to horseradish peroxidase were from Sigma–Aldrich (Germany), and anti-rat secondary antibodies were from Rockland immunochemical (Gilbertsville, PA, USA). Anti-mouse, anti-rabbit, anti-rat 488, 594, and 647 secondary fluorescent antibodies were from Thermo Fisher Scientific. IRDye800CW and IRDye680RD were from LI-COR Biotechnology. The dilutions of each antibody stock are mentioned in the appropriate “Materials and Methods” section or figure legends.

### Generation of Phosphorylation-State Specific Antibodies

Phosphorylation-state specific antibodies were generated by immunizing mice or rabbits with synthetic monomeric Aβ20-34 peptides with Ser-26 in phosphorylated (antigen sequence: FAEDVG(p)SNKGAIIGC) or non-phosphorylated state (antigen sequence: FAEDVGSNKGAIIGC) conjugated with keyhole limpet hemocyanin (KLH) as an immunogen. Hybridoma cell clones were generated and antibodies were characterized for their specificity against pSer26Aβ and npAβ peptides by indirect ELISA and WB. By protein G affinity chromatography, we purified the antibodies from conditioned media of hybridoma cell lines. Rabbit polyclonal phosphorylation state-specific antibodies were purified from the serum by double-affinity purification using pSer26Aβ and npAβ peptide. The specificity of the purified antibodies was characterized by ELISA and WB.

### Preparation of Aβ Aggregates

Aggregated Aβ1–40 variants of npAβ and pSer26Aβ peptides were prepared by dissolving the respective synthetic peptides (100 μM) in phosphate-buffered saline (PBS) and incubation at 37°C with stirring. Unaggregated Aβ was obtained immediately after suspending the peptides in PBS and stored after flash freezing in liquid nitrogen until further use. Aggregated Aβ was collected at different incubation times and also flash-frozen for further use. SDS–PAGE, Native-PAGE, WB, and Thioflavin T (ThT) fluorescence assays were carried out to confirm the unaggregated (0 h) and aggregated (12, 24, 48, 72, and 96 h) state of Aβ as previously described (Kumar et al., 2011, 2012). Fluorescence intensity was determined using a Varian Cary Eclipse fluorescence spectrophotometer. Excitation and emission wavelengths were set at 446 and 482 nm, respectively. The fluorescence intensity was measured three times for each sample and then the three readings were averaged.

### Western Immunoblotting

Synthetic peptides or brain extracts were separated on 4–12% NuPAGE gels and transferred to 0.45 μm nitrocellulose membranes. The membranes were blocked and incubated sequentially with the indicated primary and secondary antibodies and then developed by enhanced chemiluminescence using ECL imager (BioRad Inc.) or LiCOR Imaging. Quantification was performed by densitometric analysis using Quantity One software (BioRad Inc.).

### ELISA Assays

Monomeric npAβ and pSer26Aβ peptides were used as antigens for coating using PBS, pH 7.4, as coating buffer. For the antibody titer evaluation, 1 μg/ml Aβ working stock solution was prepared in PBS and kept on ice. Hundred microliter of antigen solution (npAβ and pSer26Aβ) was added per well and incubated at 4°C for 16 h. After incubation, residual liquid from the plate was removed by gently tapping the plates. Two-hundred microliter blocking buffer (1 mg/ml BSA) was added per well and incubated at 25°C for 2 h, and wells dried again. To measure the antibody titer, 5H11C10 antibody (1 mg/ml) and SA6193 antibody were serially diluted to 1:10, 1:30, 1:90, 1:270, 1:810, 1:2,430, 1:7,290 in coating buffer, added respectively to the wells and incubated at 25°C for 2 h. After the incubation, the wells were washed with 200 μl of PBS, thrice, and finally, the residual liquid was removed by gentle tapping. Hundred microliter diluted secondary antibodies (Dilution: 1:2,500; anti-mouse IgG-HRP conjugate or anti-rabbit IgG-HRP conjugate) was added to each well respectively and incubated at 25°C for 2 h. After incubation, the wells were washed thoroughly as mentioned above. One-hundred microliters of 3,3′,5,5′-tetramethylbenzidine (TMB) substrate was added to each well and the plate incubated at 25°C until sufficient blue color developed. Hundred microliter of stop solution (4 M H_2_SO_4_) was added to each well and plates were read at a Tecan plate reader at a wavelength of 450 nm and background measurement at 620 nm. Multiple readings were recorded for a single well and averaged. In each experiment, reading for a single sample was recorded in technical triplicates. Data were averaged and plotted as the mean of the readings along with standard deviation (*n* = 3).

For the detection of different concentrations of npAβ and pSer26Aβ peptides by ELISA with 5H11C10 and SA6193 antibodies, Working stock solutions of 2.5, 1.0, 0.5, 0.25, 0.1, 0.05, 0.025 ng/μl were prepared and 100 μl of stock solutions were added per well to achieve final concentrations of 250, 100, 50, 25, 10, 5, 2.5 ng/per well. One-hundred microliters of 5H11C10 and SA6193 antibody solutions (Dilution: 1:250, in blocking buffer) was added to each well. Further incubation steps and measurements were performed as mentioned above.

### Protein Extraction and Immunohistochemistry

For biochemical analysis of pSer26Aβ and npAβ variants, full-length APP (Fl-APP), and APP C-terminal fragments (APP-CTFs), whole-brain homogenates from APP/PS1ΔE9 (Tg) and non-transgenic (WT) mice were prepared as described previously (Kumar et al., 2011). APP/PS1ΔE9 mice were obtained from the Jackson Laboratories (strain # 005864; Jankowsky et al., 2004). Mice were housed under standard conditions at 22°C and a 12 h–12 h light-dark cycle with free access to food and water. Animal care and handling was performed according to the Declaration of Helsinki and approved by the local ethical committees (LANUV NRW). Briefly, for sequential Aβ extraction, brain tissue was homogenized with a douncer followed by sonication in RIPA buffer containing protease and phosphatase inhibitors. Homogenates were cleared by centrifugation at 14,000 *g* for 30 min at 4°C. After centrifugation, the resulting supernatant containing water-soluble proteins was aliquoted, saved at −80°C for further usage and the pellet was re-homogenized in 2% SDS in 50 mM Tris buffer (pH 7.3) supplemented with protease and phosphatase inhibitors followed by sonication and centrifuged as described above. The resultant supernatant SDS extract was aliquoted and stored at −80°C.

Immunofluorescence analyses of mouse brains were performed on 20 μm sagittal paraformaldehyde (PFA) fixed sections as described previously (Kumar et al., 2013, 2016). For immunofluorescence staining, the brain tissue sections were washed twice with 1× PBS and then subjected to antigen retrieval methods using reveal decloaker (Biocare Medical #RV1000) followed by permeabilization by using 0.25% triton x-100 in PBS for 30 min. Non-specific binding sites were blocked by treatment with 5% normal horse serum with 2.5% bovine serum albumin in PBS, before the addition of the primary antibodies. Mouse on Mouse (M.O.M) blocking reagent (Vector Laboratories, MKB-2213) was used for primary antibodies generated in mouse or rat (dilutions: 1:250). The primary antibodies were incubated overnight in a humid chamber at 4°C followed by incubation with an appropriate fluorescently tagged secondary antibody. After incubation, tissue sections were mounted onto slides by using VECTASHIELD Antifade mounting medium with 4′,6-Diamidino-2′-phenylindole dihydrochloride (DAPI). The z-stack images (10× magnification, 2,048 × 2,048 resolution, steps = 49, and size = 2) were acquired by using the Visitron VisiScope spinning disk confocal microscope at 10× magnification.

## Results

### Screening of Anti-pSer26Aβ Monoclonal Antibodies

**Figure 1:**
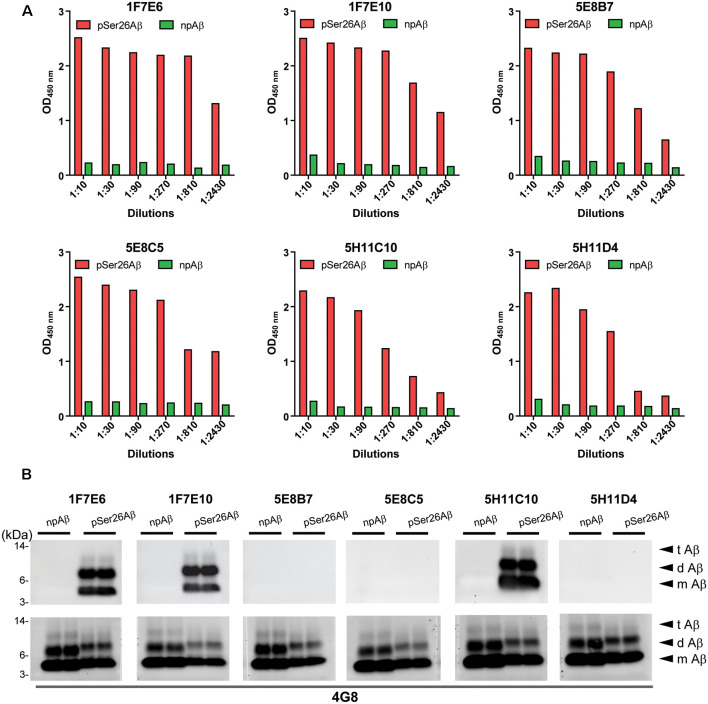
Screening hybridomas secreting anti-pSer26Aβ monoclonal antibodies. **(A)** Enzyme-linked immunosorbent assay (ELISA) assay of hybridoma supernatants with synthetic pSer26Aβ and npAβ peptides demonstrate specific reactivity against the pSer26Aβ peptides even at higher dilutions. Six hybridoma clones demonstrate a positive reaction to the pSer26Aβ peptide. **(B)** Western Blotting (WB) analysis of synthetic npAβ and pSer26Aβ peptides (200 ng) with hybridoma supernatants shows the specific detection of monomeric (m Aβ), dimeric (d Aβ), and trimeric (t Aβ) species of pSer26Aβ by three hybridoma clones. 5H11C10 demonstrates the highest reactivity. Reprobing of the blot with generic Aβ antibody 4G8 was used to detect both npAβ and pSer26Aβ peptide variants.

Hybridoma culture supernatants were screened for pSer26Aβ specific antibodies by indirect ELISA using synthetic pSer26Aβ and npAβ peptides. A total of six hybridoma clones showed a positive reaction with the pSer26Aβ peptide (Figure 1A). Then, WB was employed to further validate the monoclonal antibodies. npAβand pSer26Aβ peptides were electrophoresed and immunoblotted with hybridoma supernatants. The results showed that only three hybridoma clones among six that were positive in ELISA detect pSer26Aβ peptides by WB (Figure 1B). All three clones were highly specific for Ser26-phosphorylated Aβ and recognized monomeric, dimeric, and trimeric species of the pSer26Aβ peptide. Table 1 summarizes the class, and subclass of the hybridoma clones, demonstrating that the antibodies all belong to IgG1 (5E8B7, 5E8C5, 5H11C10, 5H11D4) or IgG2a (1F7E6 and 1F7E10), subclass with kappa light chain (Table 1).

**Table 1:** Ig classes and subclasses, and titer of monoclonal antibodies against pSer26Aβ.

Hybridoma	Class and subclass	Titer of supernatant of cell culture medium
1F7E6	IgG2a, k	>1:2,430 (pSer26Aβ); 0 (npAβ)
1F7E10	IgG2a, k	>1:2,430 (pSer26Aβ); >1:10 (npAβ)
5E8B7	IgG1, k	>1:2,430 (pSer26Aβ); >1:10 (npAβ)
5E8C5	IgG1, k	>1:2,430 (pSer26Aβ); 0 (npAβ)
5H11C10	IgG1, k	>1:2,430 (pSer26Aβ); 0 (npAβ)
5H11D4	IgG1, k	>1:2,430 (pSer26Aβ); >1:10 (npAβ)

*Starting dilution: 1:10. The titer is the highest dilution with P/N (Positive/Negative) ≥ 2.1*.

### Purification and Titer of Purified pSer26Aβ mAb 5H11C10

**Figure 2:**
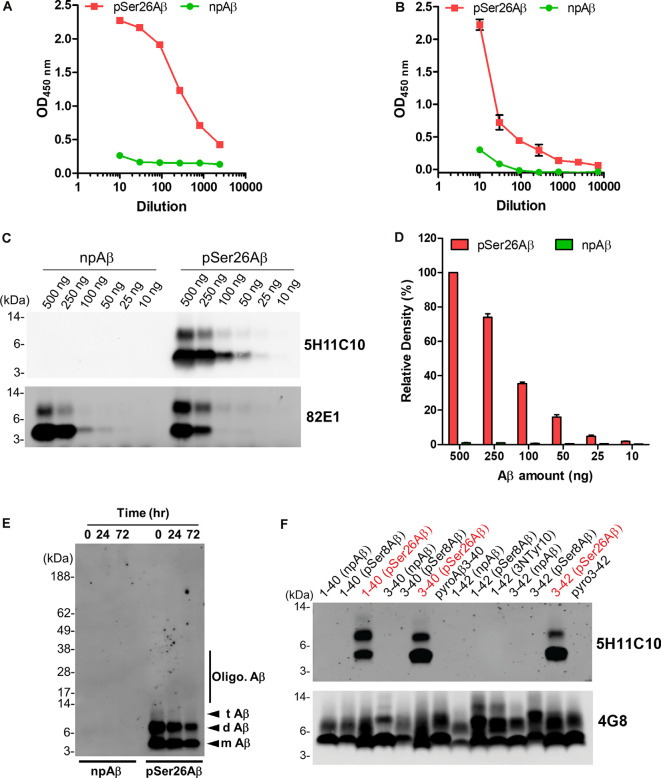
Analysis of pSer26Aβ mAb 5H11C10. **(A,B)** Titer determination by indirect ELISA of hybridoma supernatants before **(A)** and after affinity purification **(B)**. **(C)** WB analysis of different amounts of npAβ and pSer26Aβ peptides with 5H11C10 antibody (concentration: 1 μg/ml) or generic 82E1 antibody. The 5H11C10 antibody selectively recognizes as low as 25 ng of pSer26Aβ in WB and ELISA (Supplementary Figures 2A, 3A). **(D)** Densitometric quantification of the 5H11C10 antibody reactivity against various quantities of the pSer26Aβ peptide. Values indicate mean ± SD (*n* = 3). **(E)** Preparations of unaggregated (0 h) and aggregated (24 and 72 h) npAβ and pSer26Aβ peptides were electrophoresed and immunoblotted with 5H11C10 antibody. The 5H11C10 antibody specifically detects pSer26Aβ in both unaggregated and aggregated state (Supplementary Figure 4). m Aβ-monomeric Aβ; d Aβ-dimeric Aβ; t Aβ-trimeric Aβ; Oligo. Aβ-Oligomeric Aβ **(F)** Variety of non-modified, truncated, and post-translationally modified Aβ40 and Aβ42 variants were electrophoresed and immunoblotted using 5H11C10 antibody. 5H11C10 antibody specifically detects Aβ variants phosphorylated at Ser26 (written in red), whereas other modified and non-modified variants are not detected.

As 5H11C10 has the highest reactivity and titer among all of the clones in WB, this mAb was purified by fast protein liquid chromatography (FPLC) using protein G Sepharose columns. SDS-PAGE analysis shows two bands in lanes 4, 5, and 6 with a molecular weight of 50 and 25 kDa, which correspond to the molecular weights of IgG heavy chain and light chain (Supplementary Figure 1). Indirect ELISA analysis showed that the titer in hybridoma supernatants against pSer26Aβ was 2.04 × 10^7^ (Figure 2A), and purified antibody titer was 1.28 × 10^6^ (Figure 2B). WB and ELISA analysis with purified 5H11C10 antibody also demonstrated this antibody to be highly specific for pSer26Aβ peptide (Figures 2C,D and Supplementary Figures 2A, 3A). In addition to monomeric and dimeric pSer26Aβ, the 5H11C10 antibody also detected aggregated forms in a phosphorylation state-dependent manner (Figure 2E and Supplementary Figure 4). WB analysis revealed that antibody 5H11C10 also does not recognize the aggregates of non-phosphorylated Aβ when compared to the phosphorylation state-independent monoclonal antibody 82E1 (Supplementary Figure 4). Importantly, antibody 5H11C10 specifically detects Aβ variants phosphorylated at Ser26 residue and did not cross-react with other truncated and/or post-translationally modified variants of Aβ variants not phosphorylated at Ser26 residue (Figure 2F and Supplementary Figure 3B).

### Generation and Characterization of Non-phosphorylated Ser26Aβ Specific Antibodies

**Figure 3:**
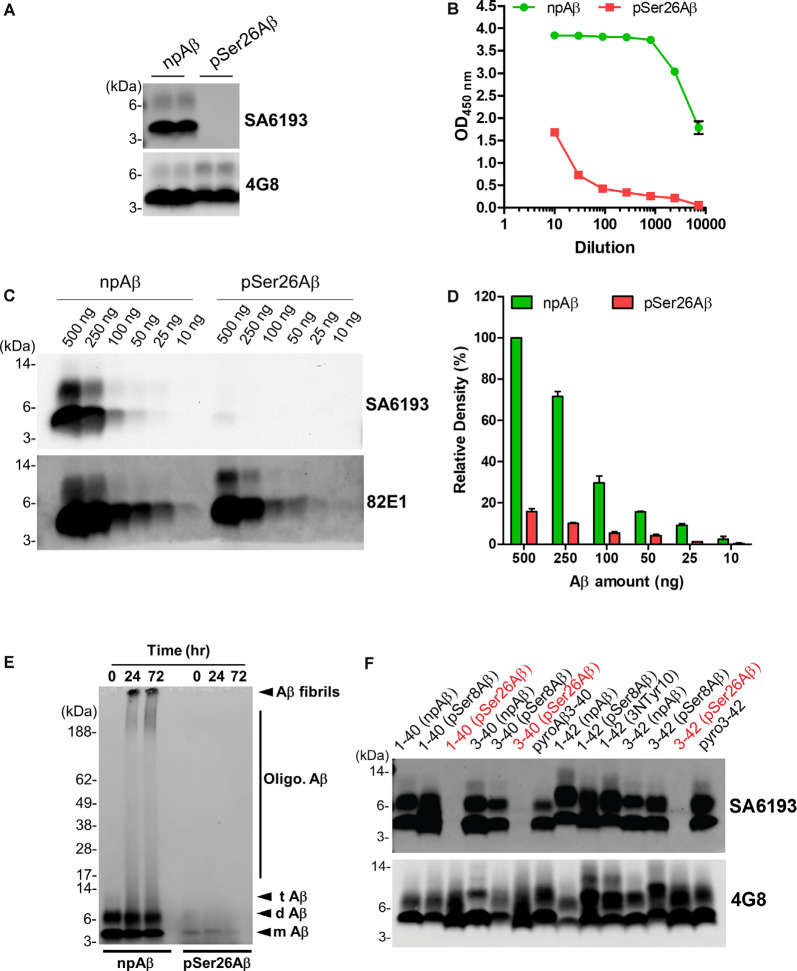
Characterization of the non-phosphorylated Ser26Aβ specific polyclonal SA6193 antibody. **(A)** WB analysis of synthetic npAβ and pSer26Aβ peptides (200 ng) with rabbit sera shows the specific detection of npAβ by SA6193 rabbit polyclonal antibody. Reprobing of the blot with generic 82E1 antibody shows the presence of both npAβ and pSer26Aβ variants. **(B)** Purified SA6193 antibody binding assessed by serial dilution of antibody on ELISA plates that were coated with npAβ and pSer26Aβ antigen. **(C)** WB analysis of different quantities of npAβ and pSer26Aβ peptides with SA6193 antibody or 82E1 antibody. The SA6193 antibody selectively recognizes npAβ monomers and dimers. SA6193 antibody selectively detects npAβ as low as 25 ng in WB **(C)** and ELISA (Supplementary Figure 2B). The membrane immunoprobed with 82E1 antibody shows the presence of both Aβ variants. **(D)** Densitometry quantification of the SA6193 antibody shows the reactivity against various quantities of the npAβ peptide. Values indicate mean ± SD (*n* = 3). **(E)** Preparations of unaggregated (0 h) and aggregated (24 and 72 h) npAβ and pSer26Aβ peptides were electrophoresed and immunoblotted with SA6193 antibody. The SA6193 antibody specifically detects npAβ in both unaggregated and aggregated state (Supplementary Figure 4). m Aβ-monomeric Aβ; d Aβ-dimeric Aβ; t Aβ-trimeric Aβ; Oligo. Aβ-Oligomeric Aβ **(F)** Variety of non-modified, truncated, and post-translationally modified Aβ40 and Aβ42 variants were electrophoresed and immunoblotted with SA6193 antibody. SA6193 antibody does not cross-react with Aβ variants that are phosphorylated at Ser26 residue but detects all of the tested non-modified (Aβ1–40 or Aβ1–42), truncated (Aβ3–40 or Aβ3–42) and modified Aβ40 and Aβ42 variants that carry other PTMs in their N-terminal regions, including phosphorylated Ser8-Aβ, nitrated and pyroglutamate Aβ (Supplementary Figure 3).

To facilitate specific detection of non-phosphorylated Ser26Aβ (npAβ) and to allow co-staining with the mouse monoclonal antibody 5H11C10, we also generated polyclonal antibodies from rabbits. Antibody SA6193 obtained from immunizations of rabbits with npAβ showed very high specificity for Aβ not phosphorylated at Ser-26 in both WB (Figure 3A and Supplementary Figure 3) and ELISA (Figure 3B). Western immunoblot analysis and ELISA of the different concentrations of npAβ and pSer26Aβ peptides showed that purified SA6193 antibody was highly specific for Aβ peptides not phosphorylated at Ser26 (Figures 3C,D and Supplementary Figure 2B). This antibody also specifically detected Aβ aggregates with Ser-26 in a non-phosphorylated state (Figure 3E). Notably, SA6193 antibody detects all of the tested non-modified (Aβ1–40 or Aβ1–42), truncated (Aβ3–40 or Aβ3–42) and modified Aβ40 or Aβ42 variants that carry post-translational modifications in their N-terminal regions, including pSer8Aβ, nitrated and pyroglutamate Aβ, but does not cross-react with Aβ variants that are phosphorylated at Ser26 residue (Figure 3F and Supplementary Figure 3B). Together, these data demonstrate that the rabbit polyclonal antibody SA6193 is highly specific for Aβ variants not phosphorylated at Ser26 residue.

### Phosphorylation State-Specific Antibodies Demonstrate the Presence of pSer26Aβ Peptides in Transgenic Mouse Models of AD

**Figure 4:**
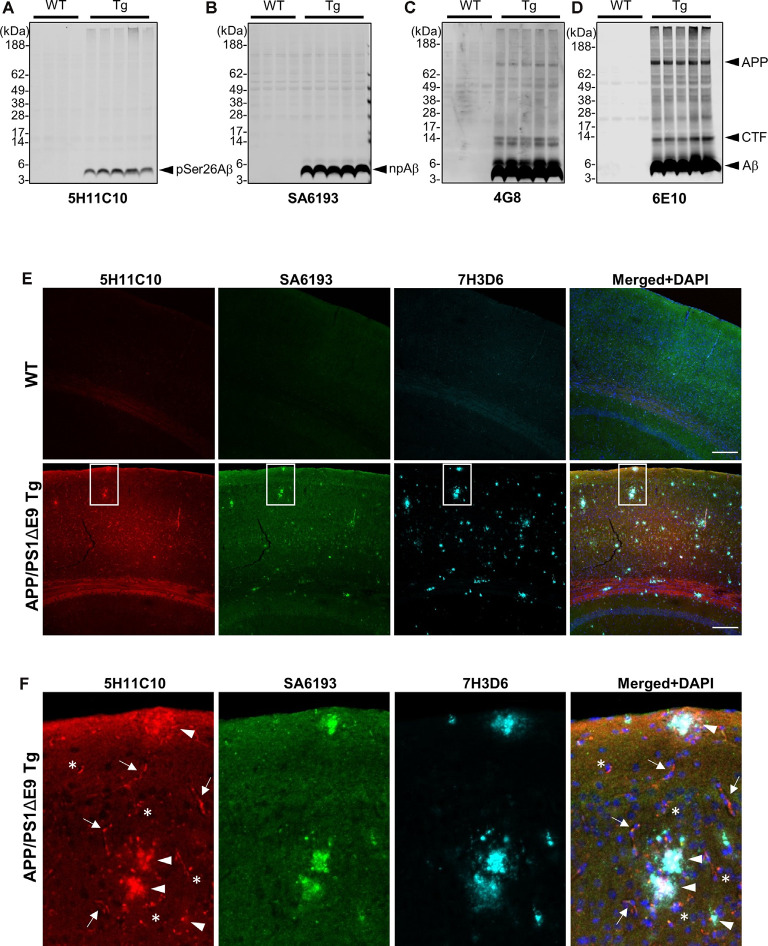
Biochemical and immunofluorescence analysis of pSer26Aβ in transgenic mouse brain. **(A–D)** WB analysis of SDS fractions of 12-months-old APP/PS1ΔE9 transgenic (Tg) and non-Tg (WT) mouse brain homogenates with 5H11C10 **(A)** and SA6193 **(B)** antibodies shows the specific detection of pSer26Aβ and npAβ peptides *in vivo*. In contrast to 4G8 **(C)** and 6E10 **(D)** antibodies, the 5H11C10 and SA6193 antibodies show no reactivity against full-length APP and APP-CTFs in transgenic mouse brain extracts, further demonstrating the specificity of these antibodies for the respective Aβ peptides with phosphorylated or unphosphorylated Ser26. **(E,F)** Immunofluorescence analysis of 12-month-old non-transgenic and APP/PS1ΔE9 transgenic mouse brain tissues with 5H11C10 and SA6193 antibodies. Representative images showing intraneuronal deposits (**F**; indicated by asterisks), extracellular amyloid plaques (**F**; indicated by arrowheads), and vascular deposition (**F**; indicated by arrows) of pSer26Aβ peptides. White boxes indicate the area in each image that is shown at higher magnification in enlarged images in panel **(F)**. Rat monoclonal 7H3D6 antibody, which is highly specific for N-terminally unmodified Aβ, demonstrates abundant staining of extracellular amyloid plaques, indicating the presence of N-terminally unmodified Aβ species starting from amino acid Asp1 predominantly in extracellular amyloid plaques (Supplementary Figure 5). Scale bar: 200 μm.

We took advantage of these antibodies to characterize the deposition of pSer26Aβ and npAβ in transgenic mouse brains. WB analysis of brain extracts from APP/PS1ΔE9 transgenic mice with 5H11C10 and SA6193 antibodies showed the presence of pSer26Aβ and npAβ peptides in SDS-soluble fractions (predominantly containing intracellular and membrane-associated Aβ) at 12 months of age (Figures 4A,B). Aβ reactivity was not detected in non-transgenic mouse brains with both antibodies. Furthermore, both antibodies did not show any reactivity against full-length APP or its C-terminal fragments in brain extracts of transgenic mice, suggesting selective phosphorylation of Ser26 after the generation of Aβ. In contrast, other commonly used monoclonal antibodies 6E10 or 4G8 also recognized the full-length APP or the APP-CTF (Figures 4C,D). Furthermore, immunofluorescence staining with the phosphorylation state-specific antibodies demonstrates the deposition of pSer26Aβ aggregates in the APP/PS1ΔE9 transgenic mouse brains (Figures 4E,F). pSer26Aβ species were faintly detected in Aβ plaques (Figures 4E,F; arrowheads). Interestingly, immunostaining revealed deposition of pSer26Aβ the cerebral vasculature (Figures 4E,F; arrows) and intraneuronally (Figures 4E,F; asterisks). Additional immunofluorescence staining with 5H11C10 together with generic Aβ antibodies further confirms the intraneuronal accumulation as well as pronounced deposition of pSer26Aβ in the blood vessels in APP/PS1ΔE9 transgenic mouse brains (Supplementary Figure 5). Detection of pSer26Aβ by the antibody 5H11C10 was efficiently blocked by pre-adsorption with synthetic pSer26Aβ, further demonstrating the specificity of this antibody in the detection of pSer26Aβ deposits (Supplementary Figure 6).

## Discussion

Here, we developed and validated novel site- and phosphorylation state-specific Ser26Aβ antibodies. The pSer26Aβ-specific mAb 5H11C10 demonstrates that pSer26Aβ is particularly accumulated in vessels and intraneuronal deposits that also contain npAβ, but much less in extracellular plaques as compared to unphosphorylated or otherwise modified Aβ species in the brain of APP/PS1ΔE9 transgenic mice.

The amyloid cascade hypothesis describes that the accumulation and aggregation of Aβ peptides into oligomeric or fibrillar structures is initiating the disease process and triggers a complex pathological cascade that ultimately leads to the development of clinical dementia (Braak and Braak, 1991; Thal et al., 2006; Haass and Selkoe, 2007; Walsh and Selkoe, 2007; Braak et al., 2011; Benilova et al., 2012; Viola and Klein, 2015; Katsnelson et al., 2016; Selkoe and Hardy, 2016; De Strooper and Karran, 2016; DeTure and Dickson, 2019; Jellinger, 2020). The accumulation of age-dependent post-translational modifications in Aβ may be contributing factors to aggregation and toxicity, and thus to the pathogenesis of AD (Thal et al., 2015; Barykin et al., 2017; Roher et al., 2017; Schaffert and Carter, 2020). Various modified Aβ species are detected in the brains of human AD patients, DS cases, transgenic AD mouse and natural animal species that develop Aβ related pathology (Saido et al., 1995, 1996; Iwatsubo et al., 1996; Russo et al., 1997; Tekirian et al., 1998; Fonseca et al., 1999; Shimizu et al., 2000; Schilling et al., 2008; Wirths et al., 2010; Saito et al., 2011; Frost et al., 2013; Kumar et al., 2013, 2016, 2020). Some of the modified species are also observed intraneuronally, years before plaque development, NFT formation, and synaptic loss (Wirths et al., 2004, 2010; Bayer and Wirths, 2010; Jawhar et al., 2011; Li et al., 2018). Post-translational modifications could accelerate the oligomerization and fibrillization of Aβ and thereby increase synaptic impairment and neurotoxicity, and the deposition in AD characteristic lesions. The differential deposition of modified Aβ variants is also associated with different stages of AD pathogenesis (Rijal Upadhaya et al., 2014; Gerth et al., 2018).

Phosphorylation of Aβ has been identified at the two serine residues within the Aβ1–40/42 peptide, Ser-8 and Ser-26 (Milton, 2001, 2005; Kumar and Walter, 2011; Kumar et al., 2011, 2016). Phosphorylation of Aβ alters its conformation, aggregation, stability, neurotoxicity, proteolytic degradation and deposition (Kumar et al., 2011, 2012; Rijal Upadhaya et al., 2014; Ashby et al., 2015; Rezaei-Ghaleh et al., 2016a, b). Immunohistochemical and immunofluorescence stainings demonstrated the occurrence of pSer8Aβ and pSer26Aβ *in vivo* in the brains of human AD patients (Rijal Upadhaya et al., 2014; Ashby et al., 2015; Gerth et al., 2018), DS cases (Kumar et al., 2020), non-human primates and canines (Kumar et al., 2018). Notably, the detection of pSer8Aβ, together with pyroglutamate modified Aβ in brain sections or brain homogenates has been recently explored to establish a staging system for AD pathology based on the sequential deposition of these modified Aβ variants during the pathogenesis of AD (Rijal Upadhaya et al., 2014; Thal et al., 2015, 2019; Gerth et al., 2018). Notably, the biochemical detection of phosphorylated Aβ species in human brains was associated with the symptomatic phase, implying that the accumulation of phosphorylated Aβ correlates with *in vitro* and *in vivo* studies that have demonstrated accelerated aggregation and increased neurotoxicity of phosphorylated Aβ peptides (Kumar et al., 2011, 2016). Thus, changes in the biochemical or biophysical properties of Aβ induced by phosphorylation may represent critical events in the pathogenesis of AD.

Site- and phosphorylation state-specific antibodies serve as important tools to investigate the spatial and temporal distribution of protein modifications in tissues or cells, and to examine their biological function (Mandell, 2003; Goto and Inagaki, 2007, 2014; Kumar et al., 2013). Our previous study demonstrated that phosphorylation at Ser-26 results in the formation of low and intermediate molecular weight soluble oligomers that remain as non-fibrillar assemblies and do not produce high molecular weight Aβ oligomers or fibrils (Kumar et al., 2016). These aggregation characteristics are reminiscent of findings on Osaka (AβE22Δ) or Dutch (AβE22Q) mutant Aβ variants that also exhibit enhanced oligomerization without fibrillization (Watson et al., 1999; Baumketner et al., 2008; Fawzi et al., 2008; Tomiyama et al., 2008; Kamp et al., 2014). These Aβ species also are detected intracellularly and in the vasculature with limited deposition in extracellular plaques (Herzig et al., 2001; Davis et al., 2004; Tomiyama et al., 2010; Kulic et al., 2012), very similar to the behavior of pSer26Aβ peptides. The amino acid residue Glu22 and Ser26 are located close to or within the β-turn motif, which plays a crucial role in Aβ monomer folding and oligomerization. Especially, the formation of the turn/bend-like structure from Gly25 to Gly29 is important for fibrillization of Aβ and is one of the earliest events in Aβ self-association. Ser26 is located at the center of the turn motif and we demonstrated that phosphorylation at Ser26 interferes with the formation of a fibril-specific salt-bridge between amino acid residues Asp23 and Lys28 (Rezaei-Ghaleh et al., 2014). The introduction of a negatively charged phosphate group at Ser26 may additionally cause intermolecular repulsive interactions that prevent or destabilize fibrillar conformations and thereby promote the formation of soluble non-fibrillar Aβ oligomers. Thus, oligomers formed by pSer26Aβ might not be incorporated in fibrillary assemblies found in extracellular plaques, but accumulate inside of neurons or the vasculature, and it will be interesting to further analyze the specific role of this Aβ species in AD pathogenesis.

Dysregulation of post-translational modifications is associated with age-related processes and contributes to age-related diseases including AD. Thus, the site- and phosphorylation-state specific antibodies against amino acid Ser26 of the Aβ peptide described here could facilitate investigations on the role of pSer26Aβ in the complex pathobiology of AD.

## Data Availability Statement

The original contributions presented in the study are included in the article/Supplementary Materials, further inquiries can be directed to the corresponding author/s.

## Ethics Statement

The animal care and handling was performed according to the Declaration of Helsinki and approved by the local ethical committees (LANUV NRW).

## Author Contributions

SK and JW conceived the study, acquired funding, and wrote the manuscript. SK, AK, ST, PJ, and FR performed experiments and analyzed data. MTH provided mouse brain tissues. All authors contributed to the article and approved the submitted version.

## Conflict of Interest

The authors declare that the research was conducted in the absence of any commercial or financial relationships that could be construed as a potential conflict of interest.
