# Supplementary figures and images for: Novel Phosphorylation-State Specific Antibodies Reveal Differential Deposition of Ser26 Phosphorylated Aβ Species in a Mouse Model of Alzheimer’s Disease
Source: Front Mol Neurosci. 2021 Jan 15;13:619639. doi: 10.3389/fnmol.2020.619639 (PMC7844098; doi:10.3389/fnmol.2020.619639)

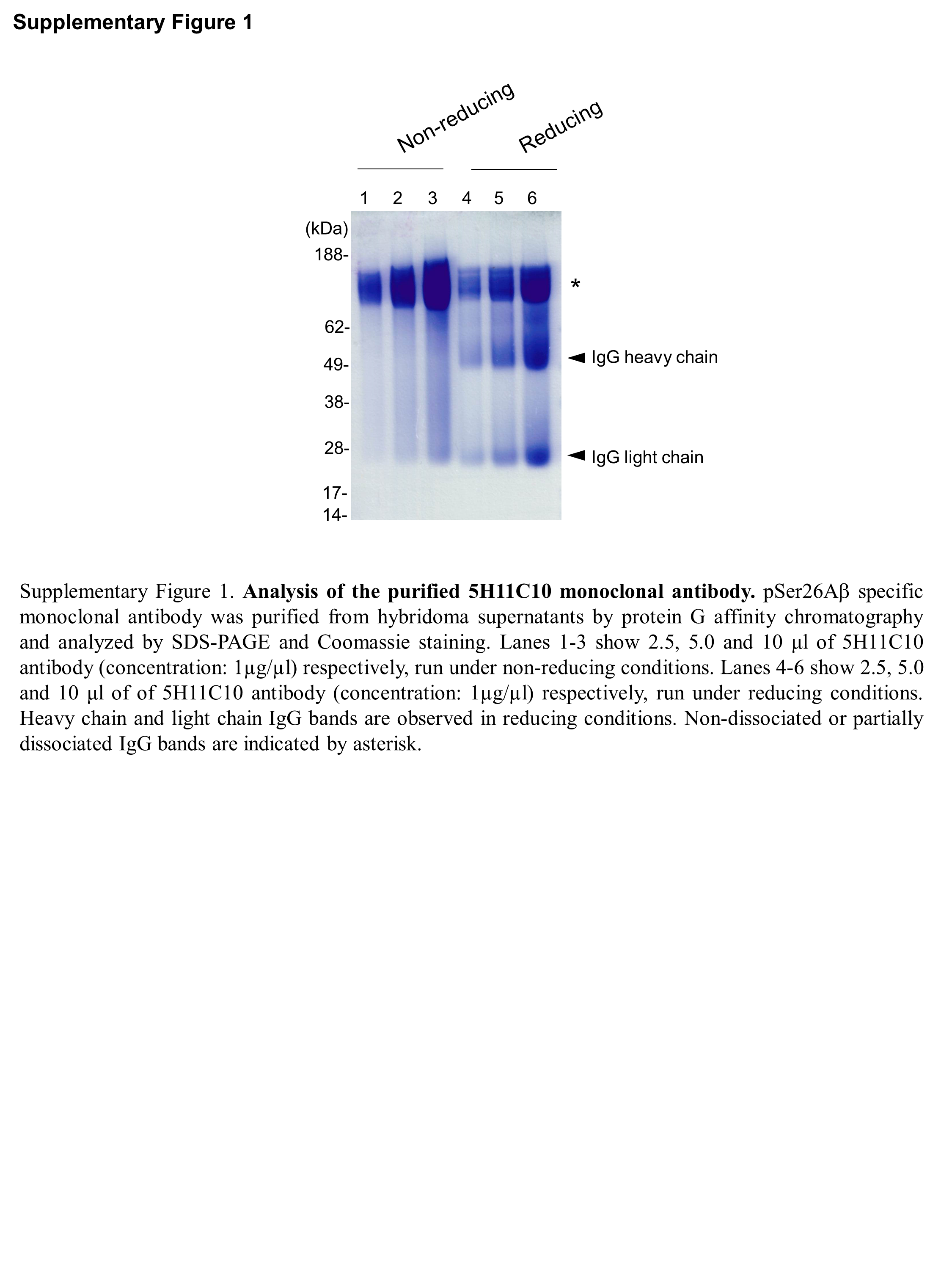

Supplement: Supplementary file 1 [file Image_1.TIF]

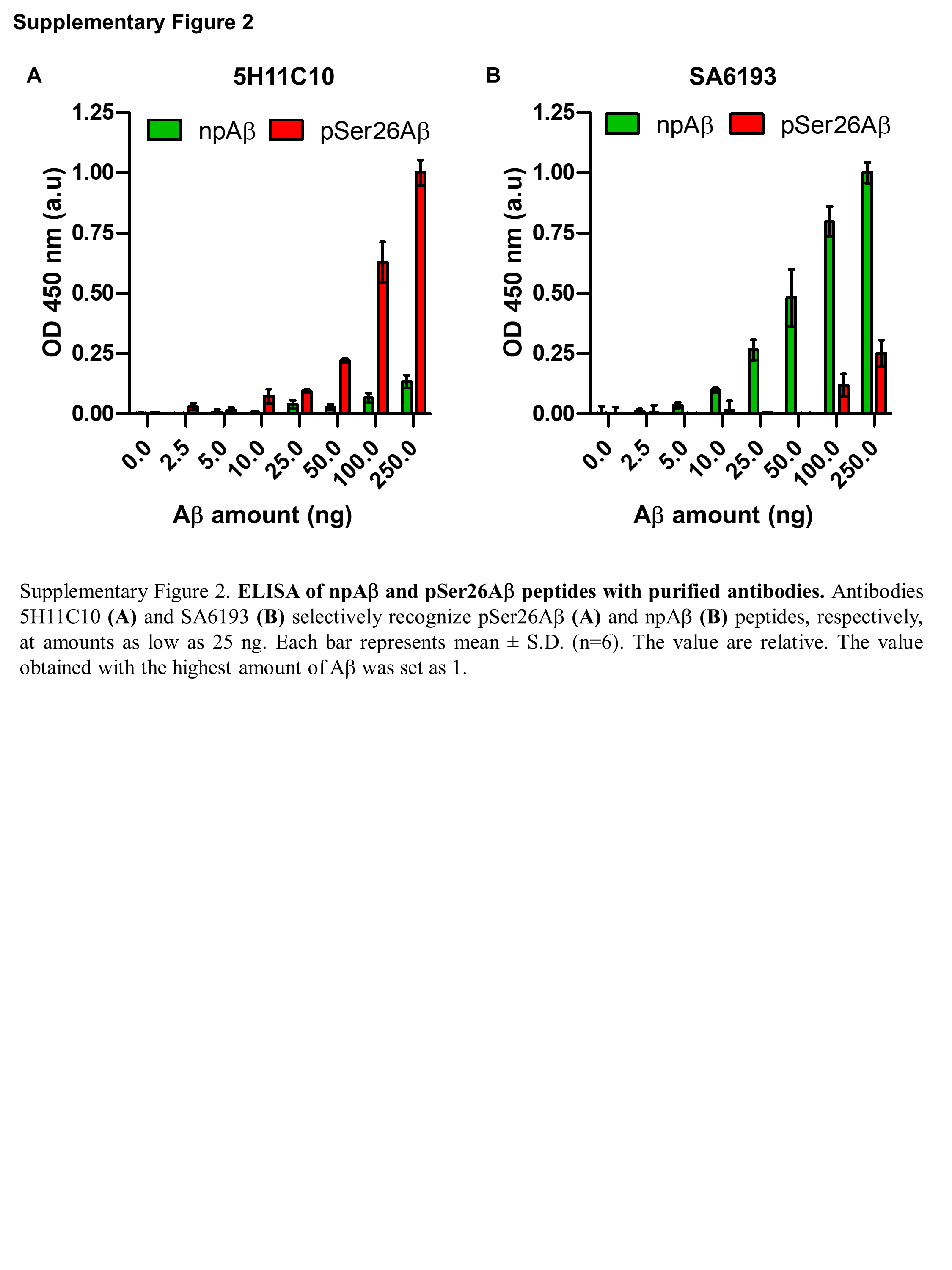

Supplement: Supplementary file 2 [file Image_2.TIF]

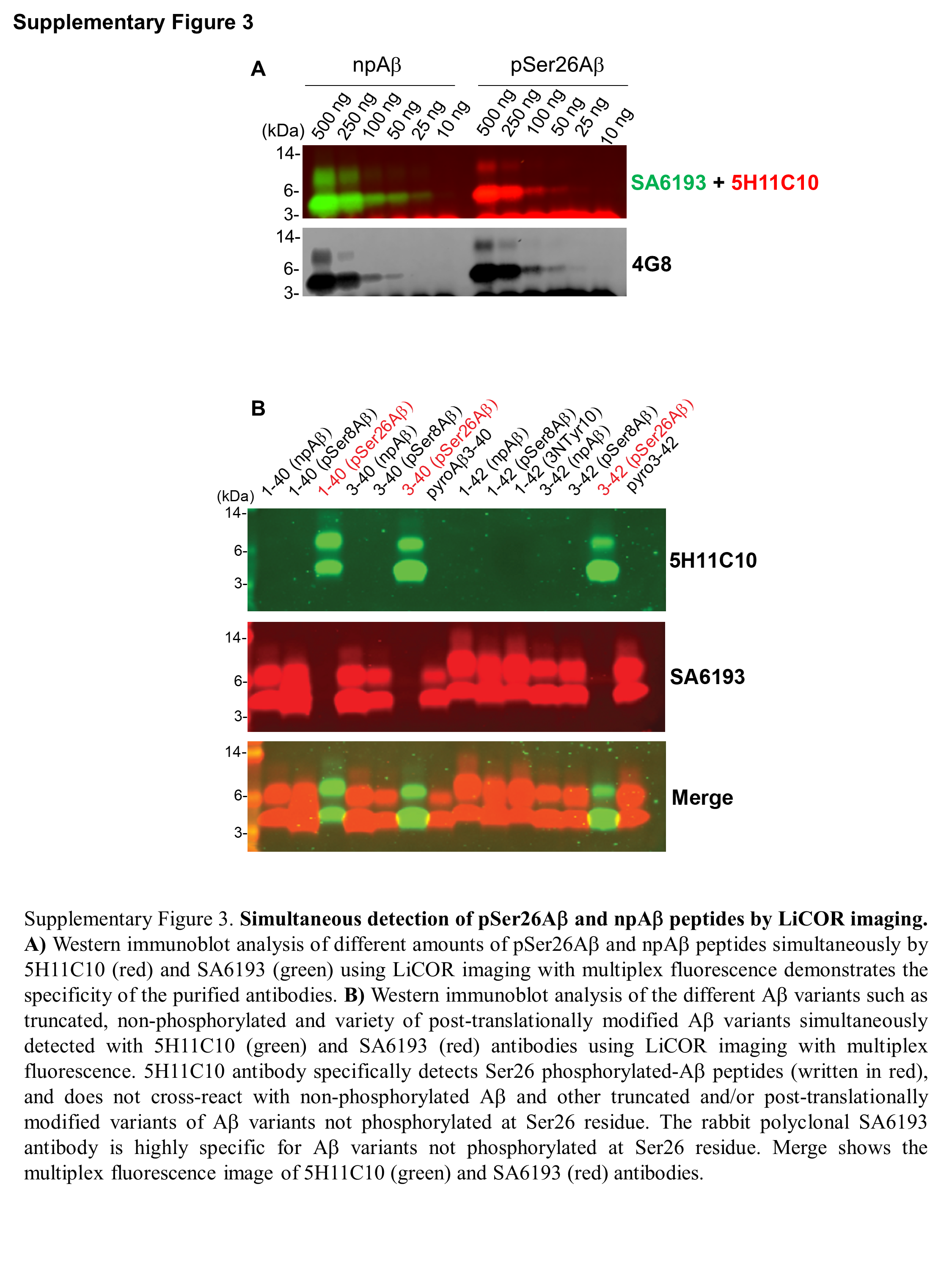

Supplement: Supplementary file 3 [file Image_3.TIF]

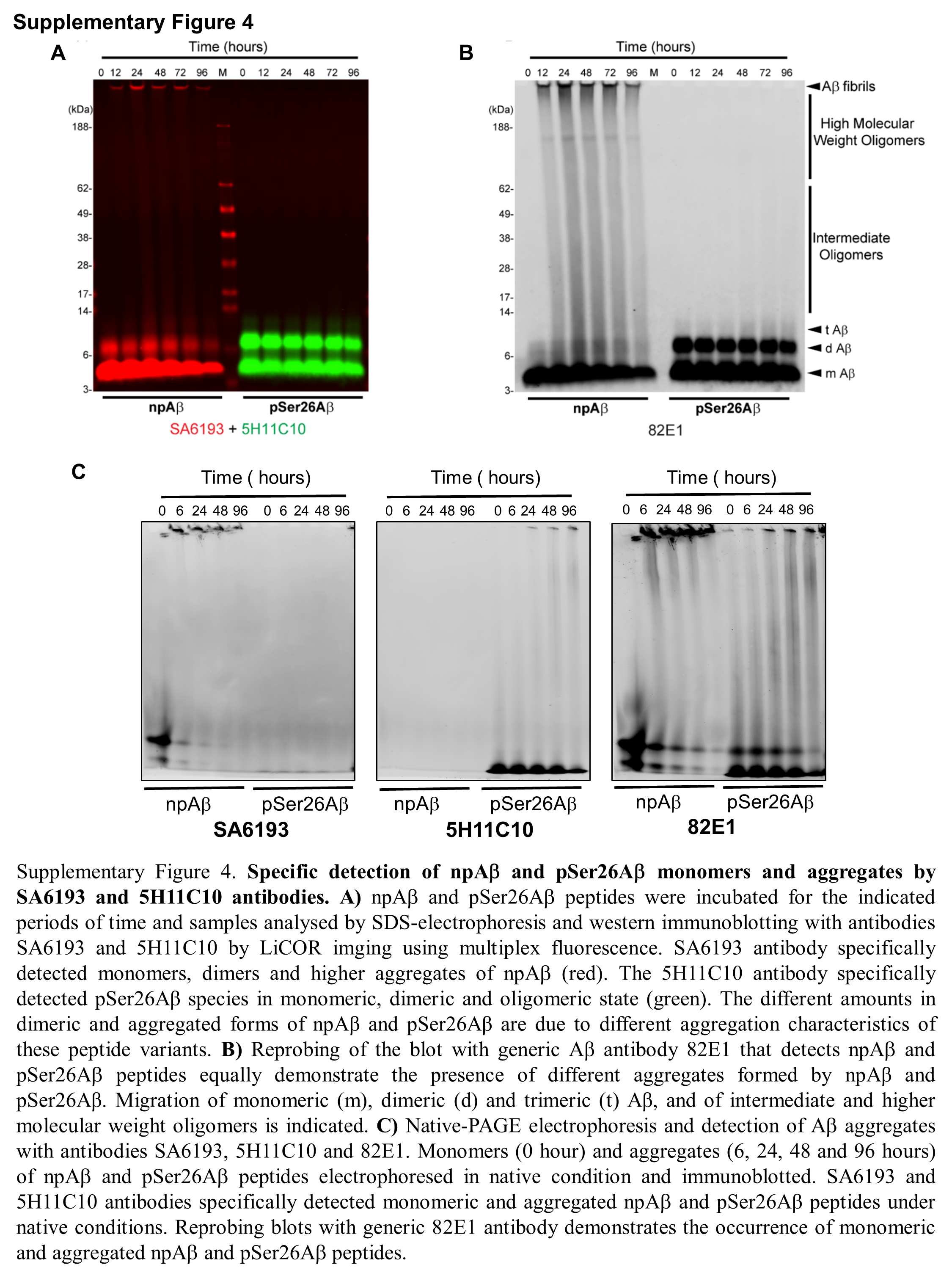

Supplement: Supplementary file 4 [file Image_4.TIF]

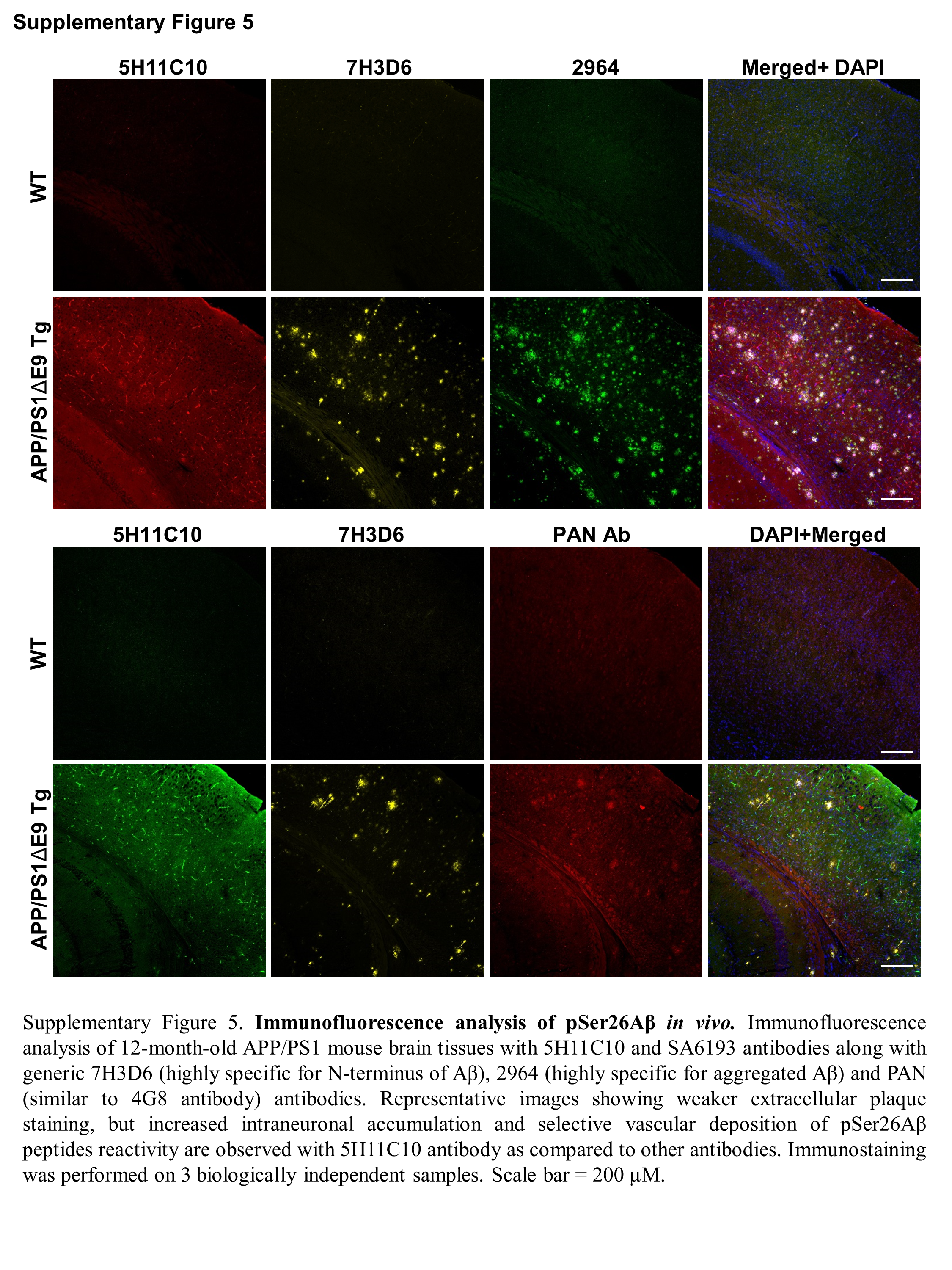

Supplement: Supplementary file 5 [file Image_5.TIF]

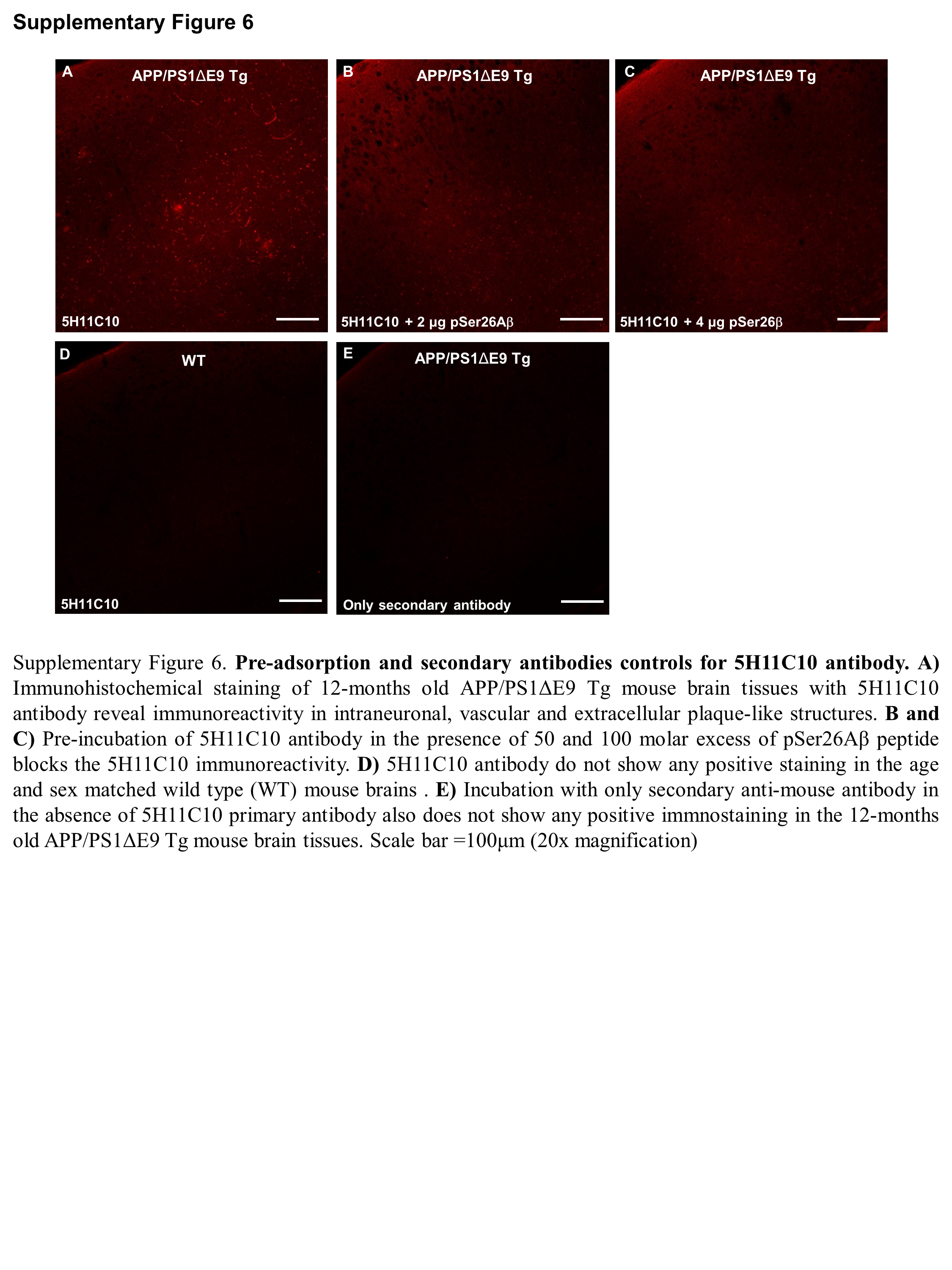

Supplement: Supplementary file 6 [file Image_6.TIF]
